# Uncertain links in host–parasite networks: lessons for parasite transmission in a multi-host system

**DOI:** 10.1098/rstb.2016.0095

**Published:** 2017-03-13

**Authors:** Josephine G. Walker, Michaela Plein, Eric R. Morgan, Peter A. Vesk

**Affiliations:** 1 School of Biological Sciences, University of Bristol, Life Sciences Building, 24 Tyndall Avenue, Bristol BS8 1TQ, UK; 2 Cabot Institute, University of Bristol, Royal Fort House, Bristol BS8 1UJ, UK; 3 Elephants for Africa, Maun, Botswana; 4 School of Biosciences, The University of Melbourne, Parkville, Victoria 3010, Australia; 5 School of Veterinary Science, University of Bristol, Langford House, Langford BS40 5DU, UK

**Keywords:** Bayesian hierarchical model, ungulates, nematodes, Botswana, bipartite, negative binomial

## Abstract

For many parasites, the full set of hosts that are susceptible to infection is not known, and this could lead to a bias in estimates of transmission. We used counts of individual adult parasites from historical parasitology studies in southern Africa to map a bipartite network of the nematode parasites of herbivore hosts that occur in Botswana. Bipartite networks are used in community ecology to represent interactions across trophic levels. We used a Bayesian hierarchical model to predict the full set of host–parasite interactions from existing data on parasitic gastrointestinal nematodes of wild and domestic ungulates given assumptions about the distribution of parasite counts within hosts, while accounting for the relative uncertainty of less sampled species. We used network metrics to assess the difference between the observed and predicted networks, and to explore the connections between hosts via their shared parasites using a host–host unipartite network projected from the bipartite network. The model predicts a large number of missing links and identifies red hartebeest, giraffe and steenbok as the hosts that have the most uncertainty in parasite diversity. Further, the unipartite network reveals clusters of herbivores that have a high degree of parasite sharing, and these clusters correspond closely with phylogenetic distance rather than with the wild/domestic boundary. These results provide a basis for predicting the risk of cross-species transmission of nematode parasites in areas where livestock and wildlife share grazing land.

This article is part of the themed issue ‘Opening the black box: re-examining the ecology and evolution of parasite transmission’.

## 1. Introduction

Management strategies for parasites could potentially be improved by incorporating additional realism into models of transmission, such as multi-host interactions and environmental effects [1]. Most pathogens and parasites can infect multiple hosts [2,3], and yet due to relative tractability, single-host single-parasite studies form much of the field of disease ecology. More recently, the impact of multi-host diseases, and particularly of emerging zoonotic diseases, such as Ebola, SARS and MERS, have led to renewed interest in understanding transmission patterns among all potential hosts [4–6]. Research directions to improve management of multi-host diseases include determining which hosts maintain the disease, as well as simply identifying all of the host–parasite interactions in a system which has not been exhaustively sampled [7].

To describe multi-host and multi-parasite systems, disease ecologists are starting to draw on concepts from community ecology [8]. Host–parasite interactions can be represented as a bipartite network (where nodes of one trophic level are linked only with nodes in a different trophic level) in the same way as other interaction systems, such as plant–pollinator, plant–herbivore or prey–predator networks [9]. This is particularly suitable for macroparasites, which can be counted and therefore their abundance incorporated into the network, as hosts with high abundance of parasites will contribute disproportionately to onwards transmission [10,11]. Macroparasites are typically aggregated, with a small proportion of the hosts infected by a large proportion of the parasites [12]. In addition, many macroparasites, and in particular the majority of the nematode species infecting ungulate hosts, are generalists, meaning that they infect multiple host species [13]. By projecting a host–parasite bipartite network into a host–host unipartite network, a ‘potential transmission network’ is constructed in which hosts are connected through shared parasites [14].

Generalist parasites link other species within an ecosystem, which may lead to apparent competition between host species. For instance, parasite-mediated apparent competition may cause exclusion of grey partridges from areas where pheasants are present [15]. Such interactions have implications for management of parasites in livestock in mixed-use systems. A better understanding of the degree of sharing of parasites between wild and domestic species could improve management strategies for parasite control in livestock in areas where grazing land is shared. For example, transmission between species may have an impact on the spread and evolution of drug-resistant parasites; wild deer in the UK were recently shown to carry anthelmintic-resistant nematodes that could be transmitted back to sheep and cattle [16].

Previous assessments of host–parasite networks are limited by the data available, which may not be representative of the full network. A recent study of 25 communities of metazoan parasites and their hosts found that very few even approached being a complete representation of the network [17]. A frequent strategy is to use species accumulation curves to assess whether parasite diversity within the data asymptotes, such as in a study of communities of nematodes in equids [18]. Alternatively, understudied species may simply be excluded from the analysis in order to prevent bias in the interpretation of rare species' interactions [13,19]. The first of these approaches seeks to assess the magnitude of undersampling; the latter seeks to limit inference to better-sampled species.

There are methods available to calculate non-biased estimators of within-community species richness which have been applied to parasite species richness within hosts; these are estimated by extrapolating data to an asymptote using data resampling methods or by estimating the proportion of rare species in the dataset [20,21]. Bayesian hierarchical models offer an alternative approach whereby the uncertainty of each possible association or link within a host–parasite network is estimated from the data using explicit assumptions about the expected distributions. In particular, zero-inflated models allow for separating zeros from the expected abundance distribution, from zeros representing true absence [22,23]. In addition, the use of random effects allows for data-scarce groups to borrow strength from data-rich groups [24], and covariates at multiple levels can be included.

In this study, we applied a Bayesian hierarchical modelling method to existing data from extensive post-mortem studies in southern Africa where nematode parasites were counted and identified. These studies, conducted over the past century, provide an excellent resource as many report estimated or exact counts for each nematode species found in each individual host from a wide range of wild and domestic ungulate species [25]. Although the researchers were interested in questions of parasite sharing between host species, they did not have the statistical tools or computing power to assess this statistically [26,27]. Here we combine data from these historical studies in order to predict host breadth and parasite diversity in a hypothetical community of ungulates and their nematode parasites, while accounting for the uncertainty inherent in the data. By assessing uncertainty within the host–parasite network, we aim to identify species from which further research would be most beneficial, and to determine whether the structure of the network is different from a network constructed from observed data only. We then use the predicted host–parasite network to project a host–host transmission network with links weighted by the number of shared parasite species. The predicted network will improve our understanding of potential transmission patterns within a multi-host multi-parasite system.

## 2. Material and methods

### (a) Data collection

We compiled data from published reports of postmortem examination of target host species in sub-Saharan Africa with the inclusion criteria that total parasite counts of each parasite species were reported for each individual host (table 1). The target host species were all wild and domestic mammalian ungulates known to occur in the case study area of Makgadikgadi Pans National Park (MPNP) in Botswana [51], which was the focus of concurrent empirical studies on cross-transmission of parasites between wild and domestic ungulates [52,53].

**Table 1.** Wild and domestic hosts included in the study, the number of individuals (*N*) and the sources of the host–parasite data.

species	scientific name	*N*	source
blue wildebeest	*Connochaetes taurinus*	5	[28,29]
bushbuck	*Tragelaphus scriptus*	15	[28–30]
Cape buffalo	*Syncerus caffer*	28	[31]
common duiker	*Sylvicapra grimmia*	20	[29,32]
gemsbok	*Oryx gazella*	7	[28,29,33]
giraffe	*Giraffa camelopardalis* (*angolensis*)	2	[29]
greater kudu	*Tragelaphus strepsiceros*	9	[28,29]
impala	*Aepyceros melampus*	46	[28,34]
red hartebeest	*Alcelaphus buselaphus*	2	[29,35]
springbok	*Antidorcas marsupialis*	72	[29,35–37]
steenbok	*Raphicerus campestris*	3	[29]
Burchell's zebra	*Equus quagga burchellii*	19	[38,39]
cattle	*Bos taurus* (*indicus*)	103	[40–44]
donkey	*Equus africanus asinus*	26	[45–47]
horse	*Equus ferus caballus*	30	[45]
sheep	*Ovis aries*	379	[48–50]

Papers were identified through a search of Web of Science with the search term ‘TOPIC:(helminth* AND Africa AND (elephant OR wildebeest OR zebra OR bushbuck OR buffalo OR cattle OR duiker OR donkey OR eland OR gemsbok OR giraffe OR goat OR kudu OR hippo* OR horse OR impala OR hartebeest OR roan OR sable OR sheep OR springbok OR steenbok OR rhino*))’. Titles and then abstracts were assessed to determine whether the paper referred to nematodes of herbivores in Africa, and full texts were used to determine if individual count data were reported. In addition, we contacted authors of papers reporting summary data from postmortem examinations of well-studied domestic species for which no individual count data had been found and manually searched available indices (from 1969 to 1973 and 1985 to 2003) from the *Onderstepoort Journal of Veterinary Research*, in which much of the parasitology work in Southern Africa has been published [25].

Parasite species names were checked against several references [54,55] for synonyms and updated where necessary. Only parasites where a binomial species name was given were included. In a few cases female parasites were identified to genus and males to species, in which case female counts were divided proportionally within an individual host to match the distribution of males. In sheep and cattle only, some tracer animals were included that were treated with anthelmintics (to clear them of gastrointestinal nematode infection) before being infected naturally by grazing for a set time period, normally four to eight weeks before slaughter. Some individual sheep, cattle and donkeys had been treated with anthelmintics up to a year prior to slaughter.

Monthly precipitation and temperature data from the time of slaughter and reported study location were acquired from the Africa Drought Monitor [56].

### (b) Model design and selection

Observations of counts of parasite species *j* in host species *i* were fit to a hierarchical model with Bayesian inference. Our model allows for explicit description of processes leading to variation in observations and estimation of the expected underlying parameter distributions.

The base model is a zero-inflated mixture model [22,23]. Observed count data *Y_ij_* are modelled as random realizations from a negative binomial distribution due to the characteristic overdispersion of macroparasite infections [57]. The negative binomial distribution is defined by probability *p_ij_* and successes *r*, which relate to the mean of the distribution, *μ_ij_*:

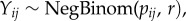


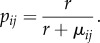
The mean abundance of parasite species *j* in host species *i, μ_ij_* combines the binary variable occurrence (whether a parasite species *j* is found in a host species *i*) *θ_ij_* with abundance of the parasite *λ_ij_* within a host species, so *μ_ij_* is 0 if *θ_ij_* = 0 and *λ_ij_* otherwise.

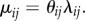
The occurrence *θ_ij_* is modelled as the outcome of a Bernoulli trial so that it equals 1 with probability *π_ij_*.

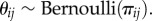
The probability of occurrence *π_ij_* is modelled with logistic regression and is determined by a host and parasite species-dependent random intercept *α_ij_*.

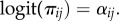
The abundance *λ_ij_* is modelled with a log link function and determined by a random intercept for each parasite species *j*, such that each parasite has abundance *β_j_* irrespective of the host and conditional on occurrence.

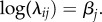
Host breadth is calculated for each parasite species *j* by summing *θ*_*ij*_ over all host species *i*, and parasite diversity is calculated for each host species *i* by summing *θ*_*ij*_ over all parasite species *j*. Models were fit using the Markov-chain Monte Carlo Bayesian modelling software JAGS v. 4.0 through the *rjags* and *R2jags* packages in R v. 3.1.1 [58–60] using the computational facilities of the Advanced Computing Research Centre at the University of Bristol.

Alternative models included random and fixed effects as explanatory variables for either the probability of occurrence (*π_ij_*) or the abundance (*λ_ij_*), which are presented in table 2. Fixed-effect covariates included individual-level variables host age, sex, treatment status, and rainfall and temperature at the time and place of slaughter; and host species-level variables feeding type (grazer, browser or mixed feeder), wild or domestic, and digestive system (ruminant or hind-gut fermenter/equid). The random-effect models explored using different groupings (e.g. by species or by genus) of host or parasite species to determine occurrence and abundance estimates.

**Table 2.** Alternative model specifications in the form of modifications to equations logit(*π_ij_*) = *α_ij_* (probability of occurrence) and log(*λ_ij_*) = *β_j_* (abundance). Modifications a–h are random effects and i–p are fixed effects. Observation level effects are indicated by index *k*, host species-level effects are indicated by index *i* and parasite species-level effects are indicated by index *j*.

model	description	new equation
a	parasite genus determines occurrence	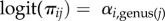
b	parasite superfamily determines occurrence	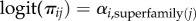
c	host and parasite determine abundance	log(*λ_ij_*) = *β_ij_*, where *β_ij_* ∼ normal(*μ_i_*, *σ*)
d	host and parasite determine abundance	log(*λ_ij_*) = *β_ij_*, where *β_ij_* ∼ normal(*μ_i_*, *σ_i_*)
e	parasite genus determines abundance	log(*λ_ij_*) = *β*_genus(*j*)_
f	parasite superfamily determines abundance	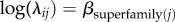
g	host genus determines occurrence	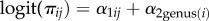
h	host genus determines occurrence	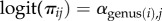
i	effect of ruminant versus equid on occurrence	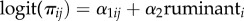
j	effect of wild versus domestic on occurrence	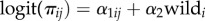
k	effect of feeding type on occurrence (ref:grazer)	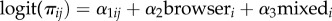
l	effect of rainfall on abundance	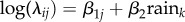
m	effect of temperature on abundance	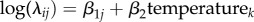
n	effect of treatment on abundance	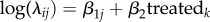
o	effect of host age category on abundance (ref:adult)	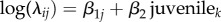
p	effect of host sex on abundance	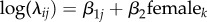

Each variation was first fitted individually, and we retained an individual effect based on whether it lowered deviance, deviance information criterion (DIC) and non-convergence (proportion of parameters with 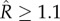) [61]. A fixed-effect parameter was considered significant and retained if the predicted 95% credible interval did not include 0. After assessment of all individual models, one retained random effect was combined with each retained fixed effect one and two at a time and the final model was selected using the same criteria, such that the model with the lowest DIC that had non-convergence <10% was selected.

Both rounds of selection were based on model runs of three chains with 50 000 steps, with the first half of each chain discarded as burn-in and the remaining samples thinned so that the final sample included 1000 steps from each of the three chains. Initial conditions were randomly selected, and non-informative priors were used for the grand means of *α* and *β*, which were drawn from normal distributions with mean 0 and precision 0.0001 [22]. Standard deviations of the grand means of *α* and *β* were drawn from weakly informative Cauchy distributions with mean 0 and precision 0.016, truncated to non-negative values [62]. The negative binomial overdispersion parameter *r* was drawn from a gamma (0.1,0.1) distribution.

Missing data for binary variables were imputed during the model fitting process as coming from a Bernoulli distribution with prior probability *p*. Sex was assumed to be *p* = 0.5, while the proportion of juveniles was calculated to match the distribution of juveniles in the sample (*p ≈* 0.35). Treatment status was only missing for domestic horses and donkeys from Theiler [45] for which we assumed *p* = 0.5. Continuous covariates (precipitation and temperature) were scaled to normal distributions with mean 0 and variance 1, and missing data were drawn from this distribution; for precipitation the distribution was truncated with a lower bound to match the data.

For the final model estimates, the selected model was run with three chains for 300 000 steps, with the first 75 000 steps of each chain discarded and the remaining samples thinned by a factor of 50 so that the final sample included 4500 steps from each of the three chains. To aid convergence hindered by negative correlation between the grand mean and standard deviation of *α_ij_*, a normal (*μ* = 0, *σ* = 50) prior truncated to the range [−8,8] was used for the grand mean of *α_ij_* and a Cauchy (*x*_0_ = 0, *γ* = 1) prior truncated to the range [0,12] was used for its standard deviation.

The host–host shared parasite network was calculated from the bipartite network by multiplying the matrix for occurrence (*θ_ij_*) by its transpose, to calculate the number of shared parasites for each pair of hosts at each step.

The final JAGS model is included in the electronic supplementary material.

### (c) Network comparison

#### (i) Host–parasite (bipartite) network

To determine how the predicted host–parasite network differs in network structure from the observed network, the median and 95% credible interval of occurrence (*θ_ij_*) from the final fitted model were compared with the data-only (unweighted) host–parasite occurrence network by calculating network-level indices connectance, links per species, cluster coefficient and nestedness using the *bipartite* package v. 2.05 in R [63,64]. Connectance is the proportion of possible links that are realized. Links per species is the mean number of links per total species (host + parasite) in the network. The cluster coefficient is per-species connectance, or the mean of the realized links divided by the possible links for each species; this is calculated for the whole network and for each trophic level [65]. The nestedness ‘temperature’ index ranges from 0 to 100 with 0 defined as maximum nestedness, where rows and columns of the network can be sorted into decreasing number of links, with each set of links exactly matching the previous or a subset of it [66].

#### (ii) Host–host (unipartite) network

To evaluate how the grouping of hosts through their shared parasites differs in the predicted and observed networks, modularity of the median and 95% credible interval, host–host networks were compared with modularity of the data-only network using the *igraph* package v. 1.0.1 in R [67]. Modularity represents clustering within the network, whereby a module within a network has many links between the nodes in that module, but few links with nodes in different modules. In this case, modules represent the groups of hosts that share a large number of parasite species. Modularity and clustering were calculated using both the *edge betweenness community* algorithm [68] and the *fast greedy* algorithm [69] for comparison, because the *edge betweenness community* algorithm tends to be more sensitive to small clusters than the *fast greedy* algorithm.

The predicted number of shared parasites in the host–host network was compared with the phylogenetic distance in molecular time between each pair of hosts by Spearman's rank correlation. Data on the phylogenetic relationship between the host species of interest was extracted from the TimeTree database [70,71] using the R package *ape* v. 3.4 [72] and visualised using the iTOL website [73].

## 3. Results

### (a) Data

**Figure 1.**
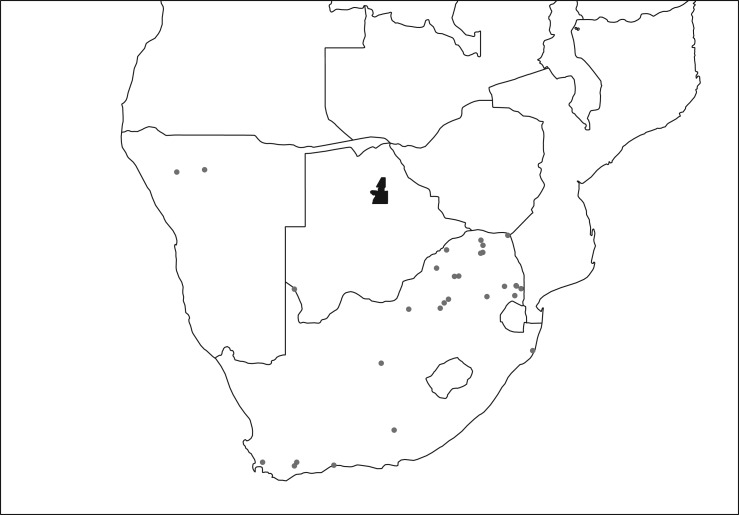
Map of data source locations. Dots represent locations of data sources; black polygon shows the location of MPNP.

The initial bibliographic search returned 923 results. After assessment of titles this list was narrowed to 176 papers, of which 21 were duplicates. Assessment of abstracts and full texts led to a final inclusion of 22 papers. No further papers were identified from the *Onderstepoort Journal of Veterinary Research*, but contacting authors led to the identification of one additional reference with data from horses, zebras and donkeys [45]. Data were available for 16 host species (table 1) infected with 124 species of parasite. The data were from a range of locations across South Africa and Namibia (figure 1).

### (b) Model selection

The results of alternative model runs are shown in tables 3 and 4. Model c, which predicts parasite abundance based on both the host and parasite, provides the best fit of the random effects models. This indicates that the mean abundance for each parasite is different within each host species, and a constant variance on the mean abundances (model c) led to a better fit than if variance was allowed to differ by host species (model d).

**Table 3.** Model selection results (phase 1). Individual effects (fixed effect, standard error of the mean (s.e.m.)) were chosen for retention in the model selection process based on minimizing non-convergence (non-conv) as well as DIC and deviance; standard deviation of the deviance (s.d.) and effective number of parameters (PD) are also presented.

model	deviance	s.d.	PD	DIC	non-conv	fixed effect (s.e.m.)
null	83 128	22	250	83 378	0.51	
a	83 113	22	245	83 359	0.06	
b	83 100	21	222	83 322	0.07	
c	81 973	27	377	82 351	0.03	
d	81 973	27	371	82 344	0.35	
e	84 168	18	160	84 328	0.52	
f	85 430	16	127	85 557	0.54	
g	83 101	21	219	83 320	0.52	
h	83 124	22	247	83 371	0.04	
i	83 111	21	224	83 335	0.44	*α*_2_ =−9.030(3.325)^a^
j	83 106	21	221	83 326	0.24	*α*_2_ =−10.200(2.940)^a^
k	83 124	22	243	83 368	0.05	*α*_2_ =−8.430(2.177), *α*_3_ =−2.418(1.654)
l	83 126	22	244	83 371	0.51	*β*_2_ = 0.068(0.046)
m	83 120	23	263	83 383	0.51	*β*_2_ =−0.114(0.050)
n	82 960	23	253	83 214	0.5	*β*_2_ =−1.552(0.102)
o	82 989	30	461	83 450	0.51	*β*_2_ =−0.949(0.107)
p	82 893	45	995	83 888	0.51	*β*_2_ = 1.180(0.133)

^a^Indicates fixed-effect parameter did not converge.

**Table 4.** Model selection results (phase 2). Model selection was based on minimizing non-convergence (non-conv) as well as DIC and deviance; fixed effect estimates with standard error of the mean (s.e.m.), standard deviation of the deviance (s.d.) and effective number of parameters (PD) are also presented. The final selected model (c + n + j) is shown in bold.

model	deviance	s.d.	PD	DIC	non-conv	fixed effect (s.e.m.)
c + i	81 959	27	373	82 332	0.17	*α*_2_ =−14.566 (3.994)^a^
c + j	81 952	27	343	82 295	0.05	*α*_2_ =−12.044 (2.696)
c + k	81 969	28	380	82 349	0.15	*α*_2_ =−8.100 (2.518)^a^, *α*_3_ =−2.449 (1.701)^a^
c + l	81 957	29	410	82 368	0.36	*β*_2_ = 0171 (0.059)
c + m	81 904	30	440	82 344	0.35	*β*_2_ =−0.307 (0.045)
c + n	81 755	27	371	82 126	0.36	*β*_2_ =−1.725 (0.116)
c + o	81 858	35	617	82 475	0.35	*β*_2_ =−0.868 (0.113)
c + p	81 168	40	807	81 974	0.35	*β*_2_ = 2.449 (0.116)
c + i + j	81 949	26	337	82 287	0.05	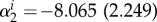 ^a^, 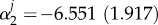 ^a^
c + n + i	81 740	27	366	82 106	0.07	*β*_2_ =−1.728 (0.114), *α*_2_ =−14.201 (3.021)^a^
**c + n + j**	**81 734**	**26**	**349**	**82 083**	**0.04**	***β*_2_ =−1.733 (0.116), *α*_2_ =−9.399 (1.817)**^a^
c + n + k	81 751	28	383	82 134	0.11	*β*_2_ =−1.728 (0.114), *α*_2_ =−8.315 (2.617)^a^, *α*_3_ =−2.647 (1.792)^a^

^a^Indicates fixed-effect parameter did not converge.

Several fixed effects also improve the model fit and/or convergence. Ruminants are predicted to have lower mean probabilities of occurrence of parasites compared to equids (model i); wild species are predicted to have lower mean probabilities of occurrence of parasites compared with domestic (model j); and browsers are predicted to have lower mean probabilities of occurrence of parasites than grazers, with no significant effect (95% credible interval not including zero) for mixed feeders compared to grazers (model k). Whether an individual animal was treated with anthelmintics is a significant factor predicted to reduce parasite abundance (model n). Rainfall has a small and non-significant positive effect (model l) and temperature has a small negative effect (model m) on abundance. Juvenile hosts are predicted to have a lower abundance of parasites than adults (model o), while females are predicted to have a higher abundance than males (model p). However, many data for age class and sex were missing, and DIC was not improved by their inclusion. Parameters that did not converge were generally subsets of *θ_ij_*, *α_ij_*, and the grand mean and standard deviation of *α_ij_*.

When the fixed-effects models were combined with model c, the best fit and best converged model combines a negative effect of treatment on parasite abundance and a negative effect of wild (versus domestic) status on overall probability of occurrence (table 4). Diagnostic trace and density plots for key parameters from the final model are shown in electronic supplementary material, figures S1 and S2. The truncation of the grand mean and standard deviation of *α_ij_* improved the model convergence, such that after the model was run for 300 000 steps, all parameters converged except for approximately 1.5% of *θ_ij_* parameters which were in the range 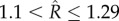. The final estimated values for the fixed effects were *β*_2_ = −1.733(0.114) and *α*_2_ = −4.049(2.054).

The fitted values match well with observed values. The model-predicted probability of occurrence is correlated with the observed prevalence of a parasite in a host (correlation = 0.767; electronic supplementary material, figure S3), and the model-predicted mean abundance 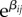 is correlated with the mean observed counts, when count = 0 are excluded (correlation = 0.997; electronic supplementary material, figure S4).

### (c) Host–parasite associations

**Figure 2.**
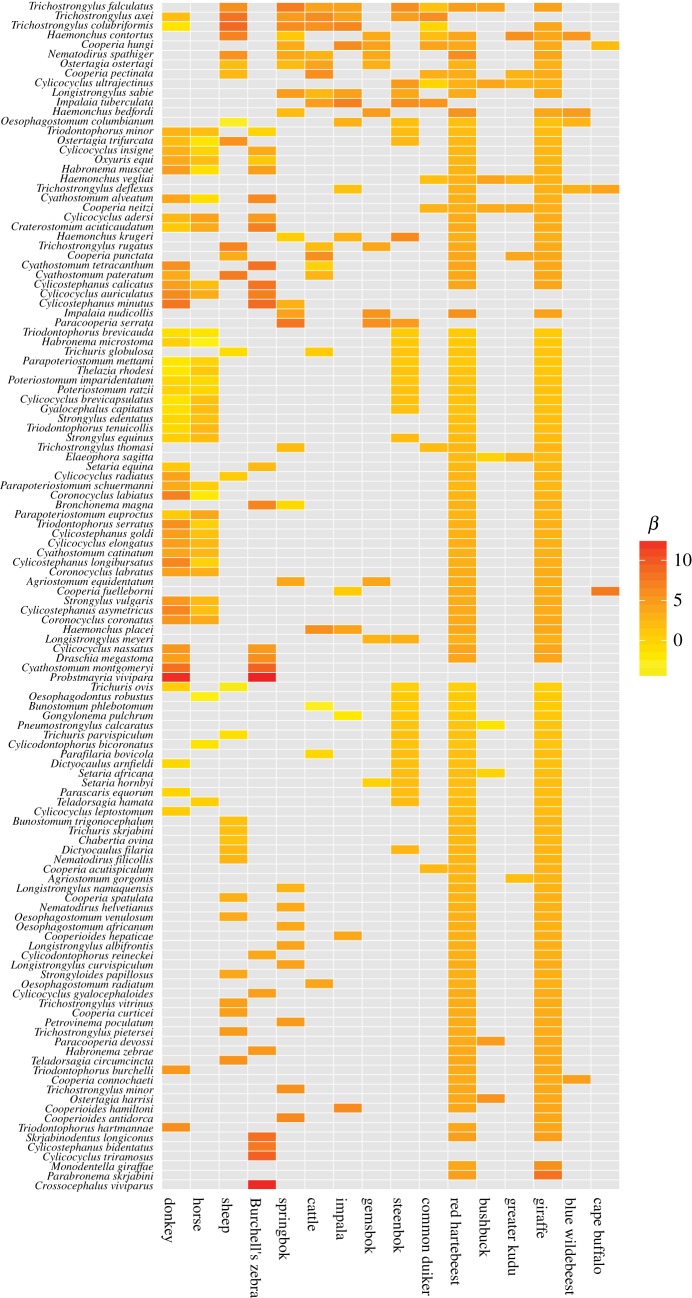
Heat map of the log predicted abundance parameter (*β_ij_*) for each host–parasite combination, from yellow (low abundance) to red (high abundance). Abundance is estimated using random effects, so host–parasite combinations for which there is little information tend to have intermediate abundance estimates. Predicted abundance is not shown for host–parasite combinations where mean *θ_ij_* < 0.05; species are ordered by summed occurrence (figure 3).

The predicted abundances (*β_ij_*) of each host–parasite combination for which *θ_ij_* is ≥ 0.05 are shown in figure 2. *Probstmayria vivipara* shows highest abundance across the board, while Burchell's zebra is the host with the highest average parasite abundance. In most cases, the estimated abundance of a particular parasite species is similar for all host species, due to random effect shrinkage. The host species for which the most data were available (e.g. sheep, donkey, horse) therefore stand out by having abundances that are different from the mean.

**Figure 3.**
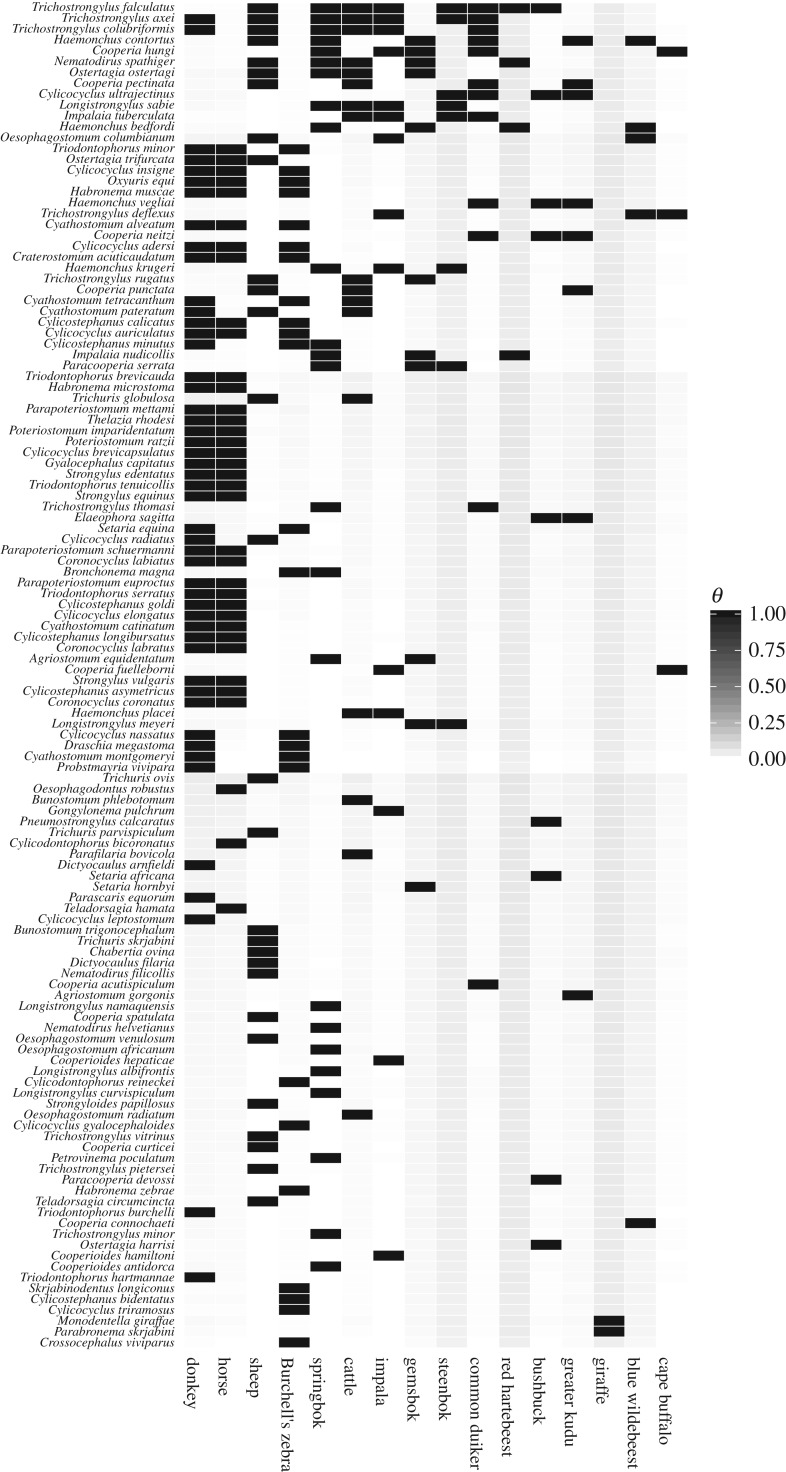
Heat map of the mean predicted occurrence (*θ_ij_*) for each host–parasite combination, ranging from 0 shown in white to 1 shown in black. Intermediate values (pale shading) indicate host–parasite combinations for which there is uncertainty in the model regarding whether the parasite occurs in that host. Species are ordered by summed occurrence. Predicted occurrence equals 1 for all observed interactions (see electronic supplementary material, figure S3).

The mean predicted probability of occurrence (*θ_ij_*) is shown in figure 3, with intermediate probabilities of occurrence found for those species for which less information was available.

**Figure 4.**
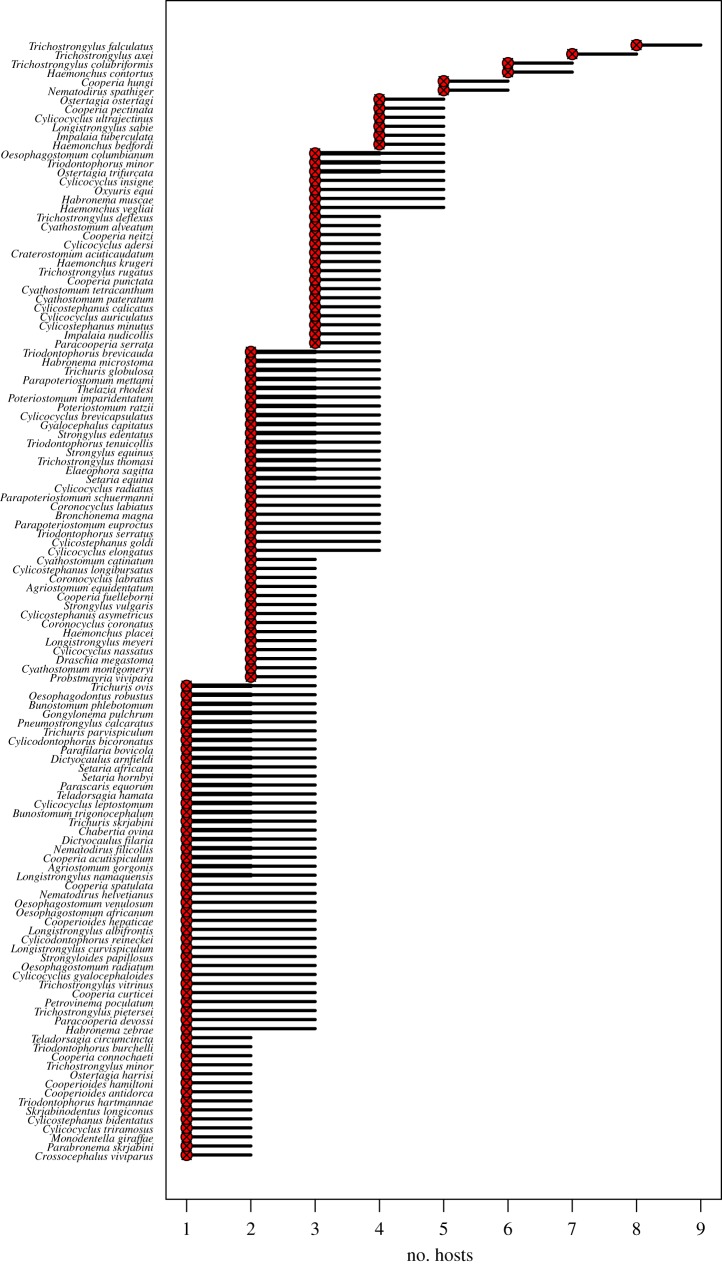
Host breadth of each parasite species predicted by the model. Circle (median), thick line (quartile range), thin line (95% credible interval). X shows observed host breadth. (Online version in colour.)

**Figure 5.**
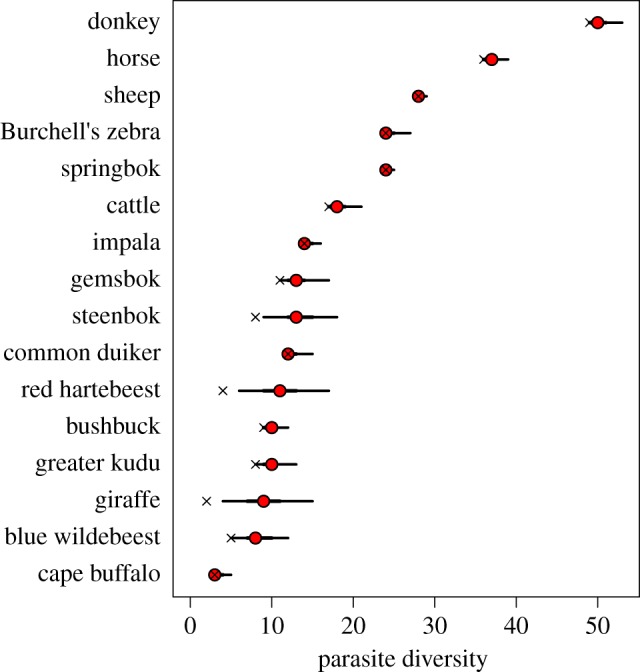
Parasite diversity of each host species predicted by the model. Circle (median), thick line (quartile range), thin line (95% credible interval). X shows observed parasite diversity. (Online version in colour.)

The model predicts that the parasite diversity within certain hosts is much higher than the observed diversity. However, it offers little in the way of prediction of which parasite species are more likely to have been missed in under-sampled hosts, as the 95% credible interval of the posterior of host breadth varies by only one or two hosts for all parasite species (figures 4 and 5).

Hosts with only two or three observations led to particular uncertainty in the predicted occurrence (figures 3 and 5, table 1); these species are red hartebeest, giraffe and steenbok. Blue wildebeest (*n* = 5) also had high uncertainty for the occurrence parameter. However, it is unclear how many samples are required to achieve a high degree of certainty. Sheep, the most sampled species, had a very narrow credible interval, while cattle, the second most sampled species, had a wider range than springbok and impala.

The probability of occurrence parameter estimates are highly bimodal (electronic supplementary material, figure S3), and links with zero observed prevalence in the data all have predicted values for the mean probability of occurrence less than 0.2. Therefore, the median predicted host–parasite network matches the observed data. However, the upper bound of the 95% credible interval (97.5th percentile) network does have approximately 25% more links than the observed network, and this translates to greater connectance, links per species, cluster coefficients and a greater nestedness index (decreased nestedness; table 5).

**Table 5.** Network-level indices for the observed network and upper bound of 95% credible interval predicted host–parasite network; lower bound and median networks are identical to the observed network.

index	observed	upper bound
connectance	0.128	0.378
links per species	1.814	5.350
cluster coefficient (total)	0.125	0.375
cluster coefficient (parasite)	0.180	0.401
cluster coefficient (host)	0.211	0.681
nestedness	18.90	28.47

### (d) Host–host networks

**Figure 6.**
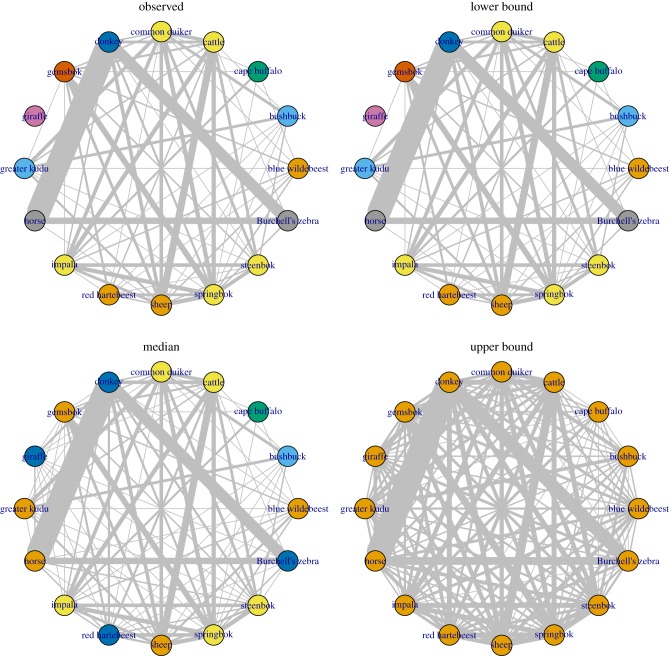
Networks weighted by the number of shared parasites for observed network, and predicted lower bound, median and upper bound networks. Nodes are coloured by *edge betweenness community*; edge width represents the weight of connection.

The predicted unipartite host networks indicate that the number of links in the observed network is underestimated (figure 6). For comparison, we present the observed network and the median predicted network, as well as the 95% credible interval networks.

The observed/lower bound network clusters into eight groups by *edge betweenness community* with modularity scores of 0.046. These host clusters are: (1) blue wildebeest, red hartebeest, sheep; (2) bushbuck, greater kudu; (3) Cape buffalo; (4) cattle, common duiker, impala, springbok, steenbok; (5) donkey; (6) gemsbok; (7) giraffe; (8) horse, Burchell's zebra. The median network clusters into five groups with a modularity score of 0.029. The clusters are: (1) blue wildebeest, gemsbok, greater kudu, horse, sheep; (2) bushbuck; (3) Cape buffalo; (4) cattle, common duiker, impala, springbok, steenbok; (5) donkey, giraffe, red hartebeest, Burchell's zebra. The upper bound network has modularity 0 and all species are in one cluster.

The *fast greedy* algorithm detects three groups in the observed and lower bound predicted networks, with modularity score 0.32: (1) blue wildebeest, bushbuck, Cape buffalo, cattle, common duiker, gemsbok, greater kudu, impala, red hartebeest, sheep, springbok and steenbok; (2) donkey, horse, Burchell's zebra; (3) giraffe. The median and upper bound predicted networks both cluster into two groups, with giraffe now included in cluster (2) with the equids, but the modularity is different: 0.26 for the median network and 0.13 for the upper bound network.

The phylogeny of the ungulate species is shown in electronic supplementary material, figure S5. The number of parasite species shared between pairs of host species is strongly negatively correlated with their phylogenetic distance: for the lower bound predicted network and observed counts (identical networks), Spearman's *ρ* = −0.59, and for the median predicted network *ρ* = −0.42, with *p* < 0.0001 for both. The parasites shared in the upper bound network are less correlated with phylogenetic distance, with *ρ* = −0.29, *p* = 0.001.

## 4. Discussion

To understand transmission of a parasite in a multi-host system, we must be able to identify which hosts can be infected, as well as the contribution of each host to transmission [11]. In some cases, a single reservoir host species may not exist, but a community of hosts can maintain transmission of a parasite when the target host on its own would not [5]. In addition, each host is likely to be infected by multiple parasites, some of which are shared with other hosts. As recent studies have shown, the structures of host–parasite bipartite networks and projected host–host networks based on shared parasites may affect patterns of transmission within an ecological community [14,74]. In this study, we estimated a host–parasite network of nematodes infecting herbivores in the MPNP, while incorporating uncertainty due to undersampling. Such a model-based approach contrasts with calculating network indices directly from field observations, which, with small sample sizes, are unlikely to be representative samples of the true distribution, for example due to a high proportion of false negatives. This method predicted that the number of parasite species infecting most of the host species is underestimated by current data (figure 5), and found that network indices from an observed host–parasite network are likely to be biased towards underestimating connectance, links per species and the cluster coefficients, while overestimating the nestedness of host–host networks of shared parasites. In particular, those host species with five or fewer individuals (table 1) showed the largest difference in predicted versus observed parasite numbers.

By using a hierarchical model structure, we were able to incorporate assumptions about parasite distribution patterns to predict whether unobserved host–parasite relationships are likely to occur. We aimed to build on known information (recorded host–parasite interactions) in a formal way to make predictions about the lesser-known parts of the system and develop quantitative evidence regarding whether absences are true absences. This method is not a magic bullet and for many of the potential links there was very little information to build on, which contributed to the difficulty in convergence of parameters related to occurrence. In particular, the model in its current form does not clearly predict which parasite species are more or less likely to occur in a given host. The mean predicted probability of occurrence for each host-parasite combination with zero observed prevalence is low (less than 0.2) and the predicted host breadth of each parasite differs from the observed host breadth by no more than two host species (figure 3; electronic supplementary material, figure S3). In those cases where the observed abundance is low, the expected distribution of a parasite within individual hosts will include many zeroes, which makes it difficult to differentiate true non-occurrence. However, the model does clearly identify those hosts about which there is the most uncertainty in their parasite fauna, and this information could be used to target additional research.

The hierarchical model structure also allows for the inclusion of covariates at different scales of the system (incorporating environmental, individual, parasite species or host species-level traits as covariates), and inferences can therefore be made from the results to influence risk assessments or management decisions at each of these ecological scales which affect parasite transmission [8]. Many of the covariates we explored in the model selection process were correlated with host–parasite associations and/or improved the fit of the model. In particular, if the individual had been treated with anthelmintics in the past year, parasite abundance tended to be lower, and wild hosts tended to have lower mean probabilities of occurrence of the parasites than domestic hosts. Incorporating additional assumptions, such as if all species in a given parasite genus were expected to have similar host occurrence, or if hosts have certain traits that are known to affect parasite fauna, would allow the model to more precisely identify expected host–parasite associations. For example, there is a large degree of uncertainty in the number of parasites expected to infect giraffe, but as they browse very high up on trees they are unlikely to be exposed to as many larvae of trophically transmitted parasites as are other host species. This expectation could be built into the model through an informative prior distribution or foraging mode effect for the probability of occurrence for giraffes, and ecological or trait-based assumptions could be incorporated for the other species with high uncertainty (red hartebeest and steenbok).

Realistic clustering of species was found in the unipartite host–host network, with equids tightly linked to each other and separate from ruminants. No predictive taxonomic information for hosts or parasites was included in the final model, but the taxonomy of the hosts was apparent in the host networks. The *fast greedy* algorithm does not identify the small clusters, but clearly groups equids separately from ruminants. The number of parasites shared by pairs of hosts was negatively correlated with phylogenetic distance, which is congruous with previous research [75–77]. This correlation was strongest in the observed data and was lower in predicted models as no assumptions were included in the model that would predict host–parasite information based on phylogeny. Similarly, uncertainty in which particular host–parasite associations were missing from the data led to a decrease in modularity of the projected network at the upper bound of the credible interval.

Although there are some parasites shared among the domestic species (figure 6), no parasite species are shared between Burchell's zebra and blue wildebeest, the two most abundant wild herbivores in the study area [51]. Both of these species migrate and share the same grazing land [78], potentially mitigating transmission of each other's parasites. On the other hand, the strong links between Burchell's zebra and domestic horses and donkeys, and between certain species of wild and domestic ruminant, indicate that there is potential for a high degree of transmission of parasites between wild and domestic species.

The data used in this study were drawn from an extensive history of research into parasites in southern Africa [25]. Another recent study used data on tick identifications from a similar long-term dataset to examine host-generalism in ticks of mammals [79]. A primary limitation of the data is that we have assumed that the host–parasite associations of southern Africa as a whole are the same as in the region of Botswana from which the set of relevant hosts was selected. Therefore, the data do not differentiate between the potential and realized niche of a parasite, as the barrier preventing infection of a particular host species may be geographical rather than biological. Despite the limitations of using data gathered for a different purpose, historical datasets provide a valuable resource, particularly where postmortem sampling is necessary for data acquisition. Currently, postmortem sampling of wildlife, and particularly of rare or endangered species, is necessarily opportunistic [28]. As a result, it may not be possible for sampling efforts for nematodes to focus on the most under-sampled species identified in this study (red hartebeest, giraffe and steenbok), or even on those species for which no data were available (with the exception of missing domestic species such as goats). A non-invasive genetic method for identifying nematode communities from faecal samples has recently been demonstrated in African buffalo [80]. Genetic barcoding of parasites would provide an additional benefit in the form of evidence as to whether a parasite species identified in different host species is the same strain, as has been assessed for *Haemonchus contortus* in ungulates in Europe [81], and would remove the biases probably introduced by morphological identification of parasites, such as the presence of cryptic species. Genetic barcoding of hosts may also reveal cryptic species; the phylogeny of African ungulates is still an active area of research, for example, bushbuck have recently been proposed to be two species [82–84]. The Bayesian hierarchical modelling method used here could be applied to genetic groups from sequenced data rather than morphological identification, for parasites and/or hosts.

Building a network that identifies areas of uncertainty in host–parasite associations, as we have done here, is an important first step towards understanding transmission in a multi-host, multi-parasite system. By examining a community of generalist parasites and their hosts rather than single-host, single-parasite systems, we are better prepared to untangle the impact that alternative hosts may have on transmission [8]. The hierarchical modelling method used in this study to predict unobserved links in host–parasite networks, in combination with more details on the abundance of hosts and the degree of overlap in grazing, could be used to predict the extent of mitigation or amplification of transmission by co-grazing species.

## Supplementary Material

Code and Supplementary Figures

## Supplementary Material

Raw Data

## Data accessibility

The code supporting this article has been uploaded as part of the electronic supplementary material. All data included are from previously published work, and the combined dataset used here has been uploaded as part of the electronic supplementary material.
