# Supplementary material for: Uncertain links in host–parasite networks: lessons for parasite transmission in a multi-host system
Source: Philos Trans R Soc Lond B Biol Sci. 2017 Mar 13;372(1719):20160095. doi: 10.1098/rstb.2016.0095 (PMC5352821; doi:10.1098/rstb.2016.0095)
Supplement: Code and Supplementary Figures [file rstb20160095supp1.pdf]

Code and Supplementary Figures

JAGS Code for Final Model

```
model
{
  for (i in 1:Nobs)
  {
      count[i] ~ dnegbin(Pp[i], r)
      Pp[i] <- r/(r+mu.eff[i])
      mu.eff[i] <- lambda[i]* use[host.sp[i], par.sp[i]]
      log(lambda[i]) <- beta[host.sp[i],par.sp[i]] + beta_t*treated[ind[i]]
  }

  for (n in 1: Nhost)
  {
      for (j in 1:Npar)
      {
          use[n, j] ~ dbern(p[n, j])
          logit(p[n, j]) <- max(min(alpha[n, j] + alpha_d*wild[n],99),-99)
          alpha[n, j] ~ dnorm(mn[1], prec[1])
          beta[n,j] ~ dnorm(sp.beta[j],prec.beta)
      }
      PD_host[n]<- sum (use[n, ])
  }

  for (j in 1: Npar)
  {
      sp.beta[j] ~ dnorm(mn[2], prec[2])
      HB_invert[j] <- sum(use[, j])
  }

  for (k in 1:2)
  {
      prec[k] <- pow(sd[k], -2)
  }

  mn[1] ~ dnorm(0, .02)T(-8,8)
  mn[2] ~ dnorm(0, .0001)
  sd[1] ~ dt(0, 1, 1)T(0,12)
  sd[2] ~ dt(0, .016, 1)T(0, )

  prec.beta <- pow(sdb,-2)
  sdb ~ dt(0, .016, 1)T(0, )

  beta_t ~ dnorm(0, 0.0001)
  alpha_d ~ dnorm(0, .0001)

  r~dgamma(0.1,0.1)

  hosts <- use[,] %*% t(use[,])
  parasites <- t(use[,]) %*% use[,]
}
```

© The Authors under the terms of the Creative Commons Attribution License http://creativecommons.org/licenses/by/3.0/, which permits unrestricted use, provided the original author and source are credited.

Supplementary Figures

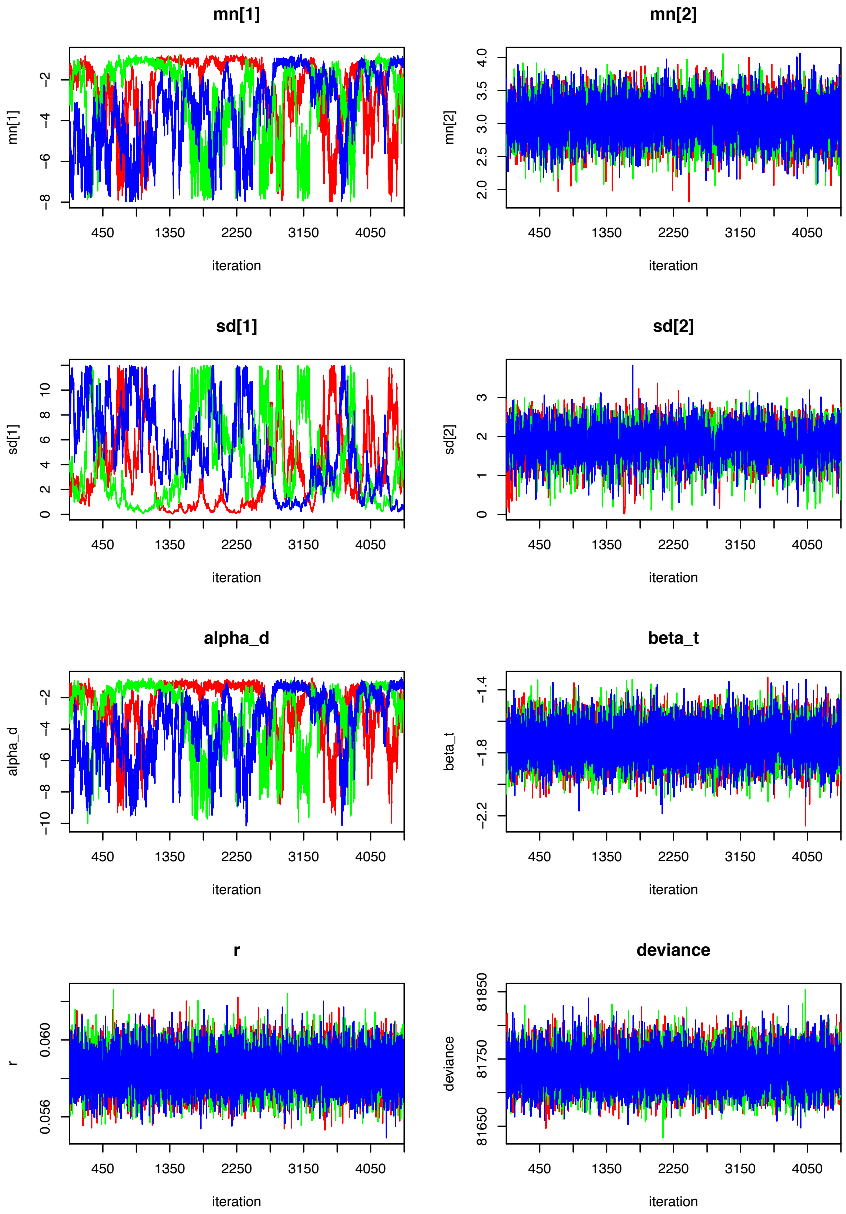

Supplementary Figure 1: Trace plots for parameters of interest showing 4500 iterations included for each of 3 chains (blue, red, green) after burn-in and thinning. See final JAGS code in online supplementary material: mn[1] is grand mean of α parameters, mn[2] is grand mean of parasite-species level β parameters, sd[1] is standard deviation of the grand mean of α parameters, sd[2] is standard deviation of the grand mean of β parameters, alpha_d is the fixed effect parameter for wild species in the occurrence sub-model, beta_t is the fixed effect parameter for anthelmintic treatment in the abundance sub-model, r is the shape parameter for the negative binomial distribution, and deviance is the overall model deviance at each iteration.

© The Authors under the terms of the Creative Commons Attribution License http://creativecommons.org/licenses/by/3.0/, which permits unrestricted use, provided the original author and source are credited.

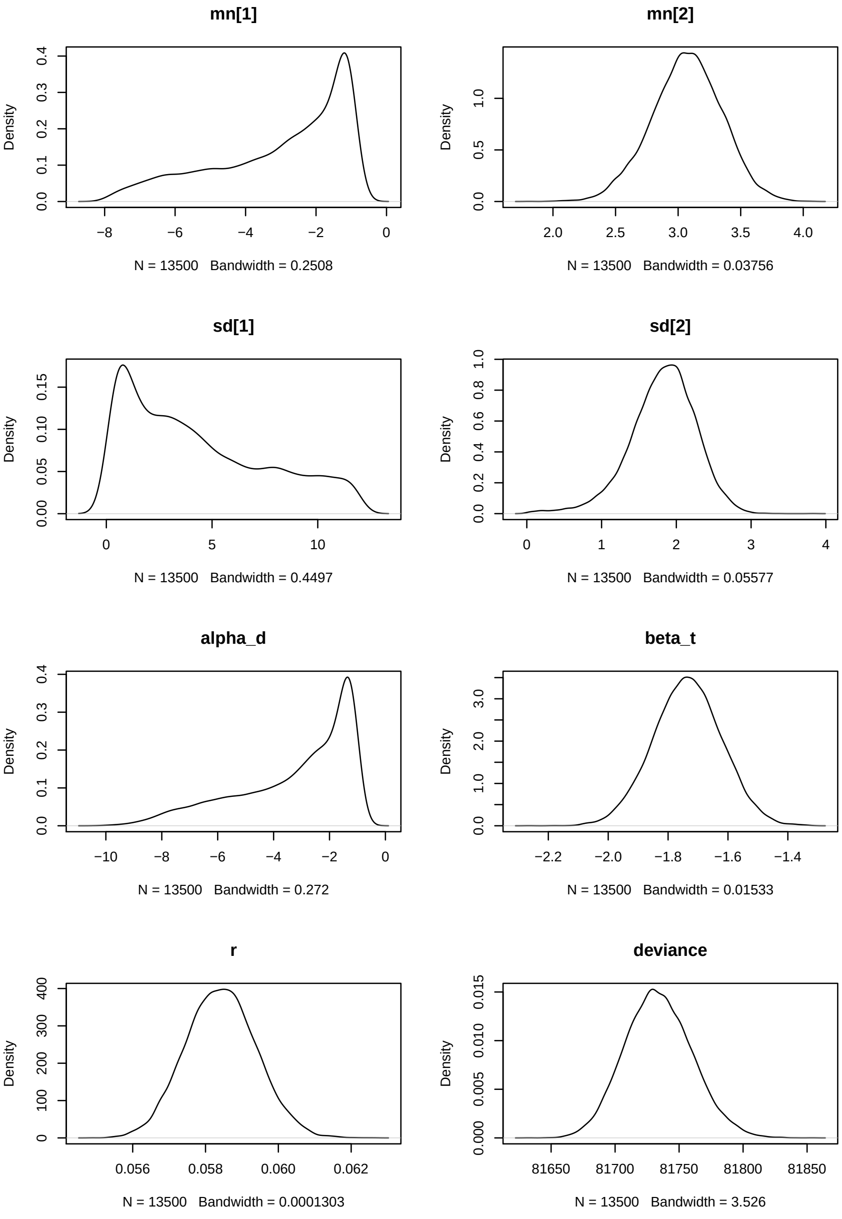

Supplementary Figure 2: Density plots for parameters of interest showing distribution from 13500 iterations (3 chains). See final JAGS code in online supplementary material (above): mn[1] is grand mean of α parameters, mn[2] is grand mean of parasite-species level β parameters, sd[1] is standard deviation of the grand mean of α parameters, sd[2] is standard deviation of the grand mean of β parameters, alpha_d is the fixed effect parameter for wild species in the occurrence sub-model, beta_t is the fixed effect parameter for anthelmintic treatment in the abundance sub-model, r is the shape parameter for the negative binomial distribution, and deviance is the overall model deviance at each iteration.

© The Authors under the terms of the Creative Commons Attribution License http://creativecommons.org/licenses/by/3.0/, which permits unrestricted use, provided the original author and source are credited.

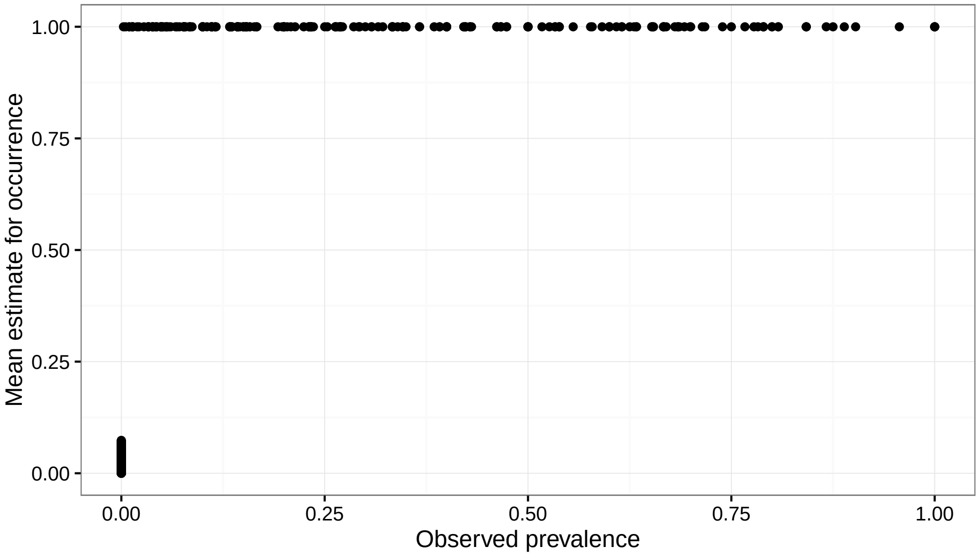

Supplementary Figure 3: Model-predicted occurrence versus observed prevalence demonstrating model fit to data. Most interactions (1730/1984) have observed prevalence = 0.

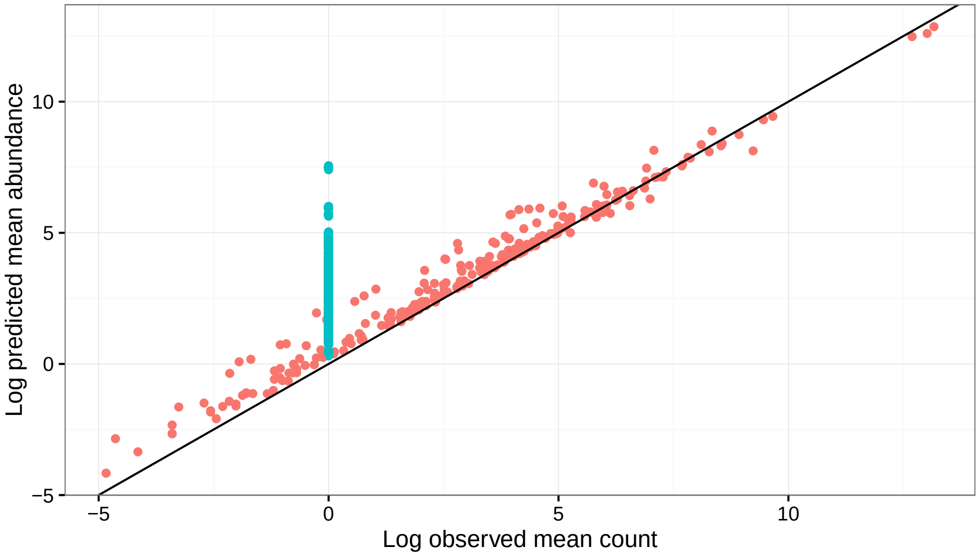

Supplementary Figure 4: Model-predicted abundance versus observed mean counts demonstrating model fit to data. Blue dots are abundance for mean count 0 (not logged). Red dots are log mean counts > 0. Black line shows $y = x$.

© The Authors under the terms of the Creative Commons Attribution License http://creativecommons.org/licenses/by/3.0/, which permits unrestricted use, provided the original author and source are credited.

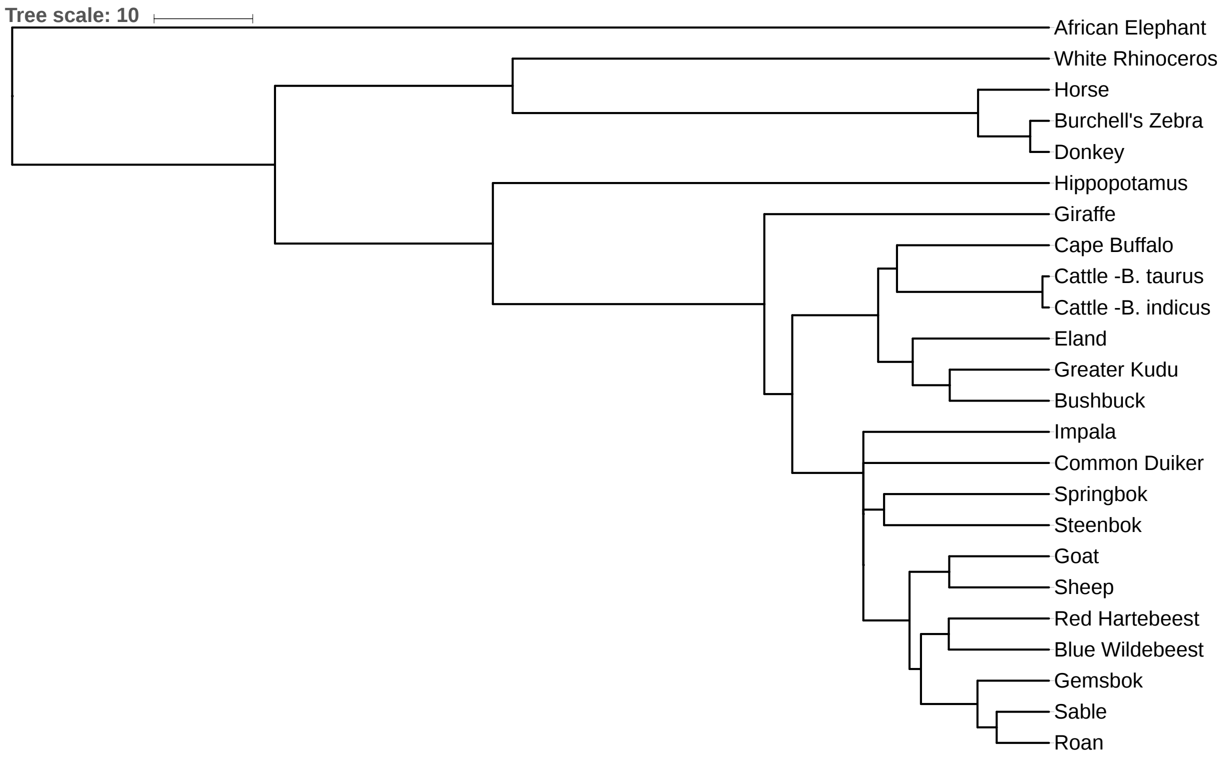

Supplementary Figure 5: Phylogenetic tree showing the evolutionary relationship between host species studied, with data from [70,71]. Branch lengths are proportional to time since divergence (in millions of years).

© The Authors under the terms of the Creative Commons Attribution License http://creativecommons.org/licenses/by/3.0/, which permits unrestricted use, provided the original author and source are credited.
